# Supplementary material for: Structural variants exhibit widespread allelic heterogeneity and shape variation in complex traits
Source: Nat Commun. 2019 Oct 25;10:4872. doi: 10.1038/s41467-019-12884-1 (PMC6814777; doi:10.1038/s41467-019-12884-1)
Supplement: Supplementary file 1 — Supplementary Information [file 41467_2019_12884_MOESM1_ESM.pdf]

Supplementary materials

Structural variants exhibit widespread allelic heterogeneity and shape variation in complex traits

Mahul Chakraborty, J.J. Emerson, Stuart J. Macdonald, Anthony D. Long

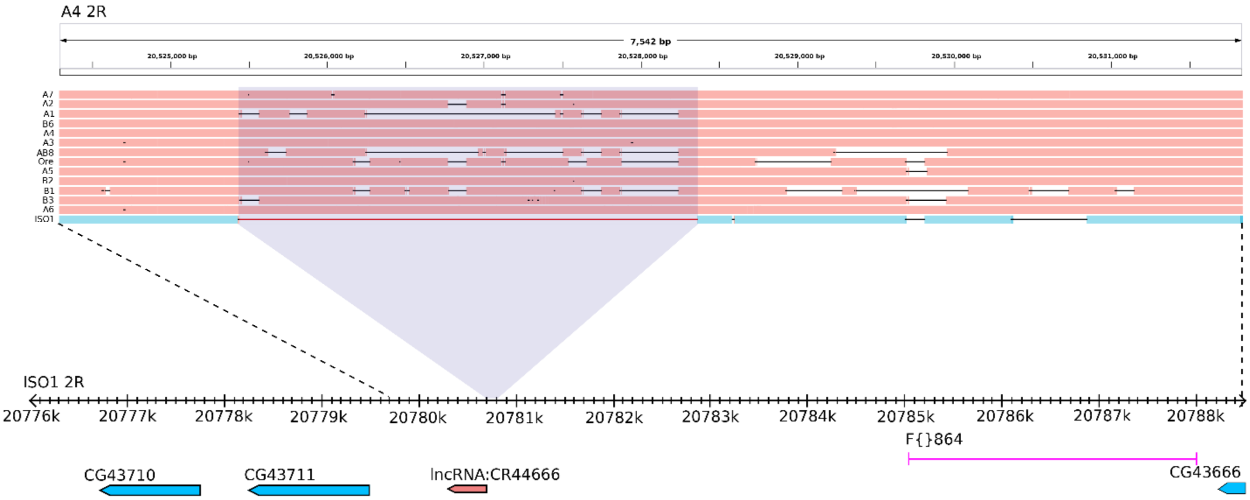

Supplementary Figure 1. Alignment of the founder genomes and the reference genome to the A4 (top) reference sequence to show the 2R euchromatic gap[1]. The gap (shaded region) in the 2R assembly of ISO1 is spanned in all of the sequenced strains described here. The gap falls within a repetitive region and harbors SVs in several founder strains. The alignment showed here corresponds to the genomic region in ISO1 marked by dotted lines.

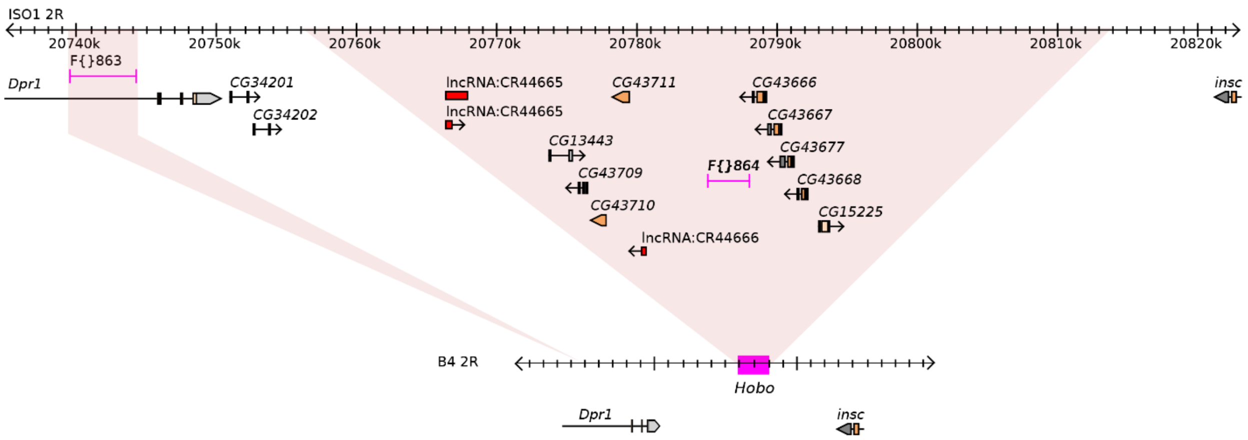

Supplementary Figure 2 The 2R euchromatic gap in ISO1 and 50Kb sequence flanking it are missing in B4. The 58kb deleted sequence harbors several functional, but presumably non-essential, genes. The deleted sequence is replaced by a ~2kb *Hobo* TE fragment in B4.

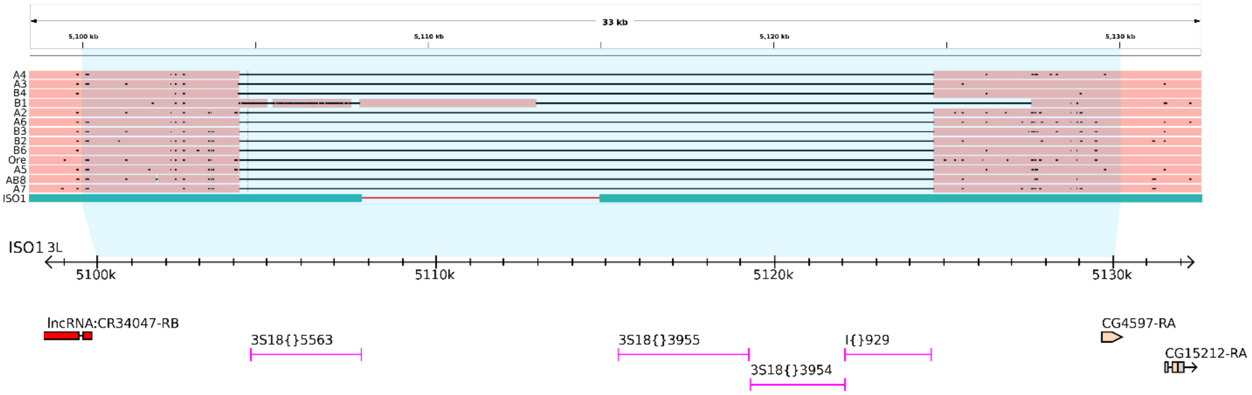

Supplementary Figure 3. Alignment of de novo founder genome assemblies to ISO1 reference genome showing the absence of the ISO1 euchromatic gap on 3L in the other assemblies. The ISO1 gap is due to duplication of a TE which is private to the ISO1 strain[1]. The alignment gap is due to the absence of the TEs (pink lines) in the new sequenced strains.

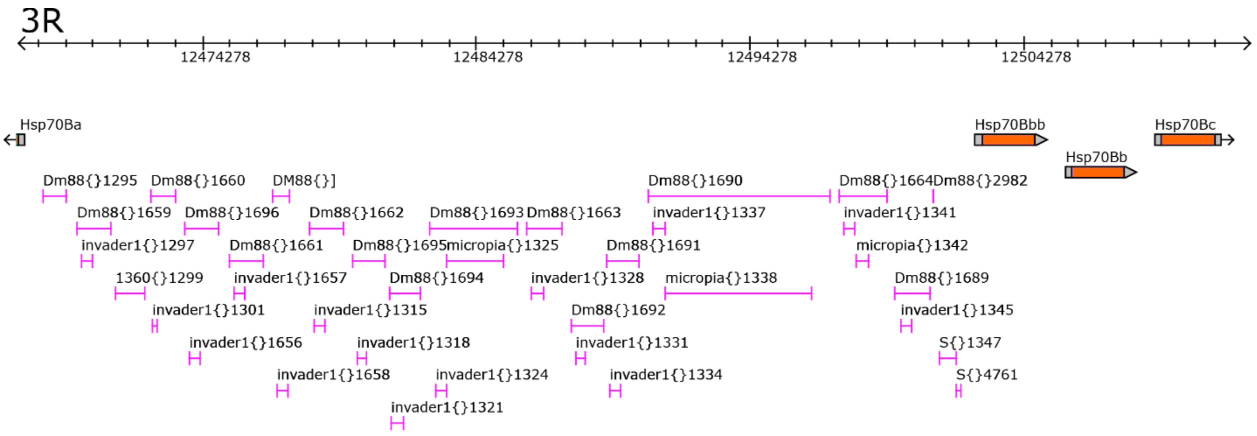

Supplementary Figure 4. A genomic region in ISO1[2] showing the TE insertion (pink lines) hotspot on chromosome 3R visible in Fig. 1c.

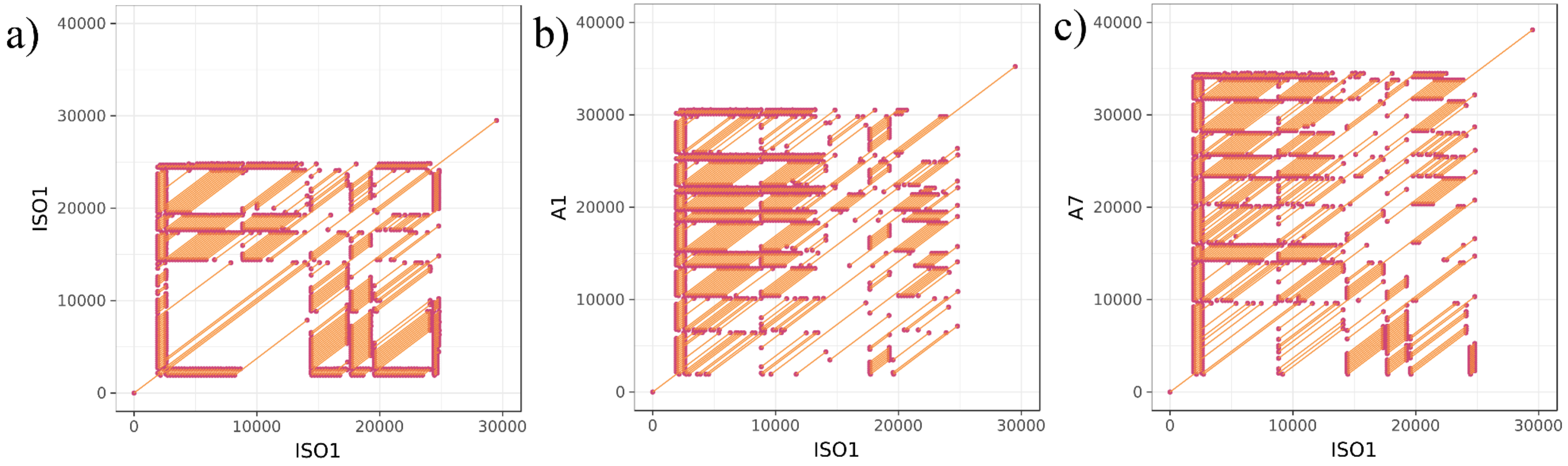

Supplementary Figure 5. Alignment dot plot of the ISO1 genomic region (3L:7667000-7696500) harboring mis-annotated SVs in A1 and A7. The dots represent alignment start and end, whereas the line connecting two dots represent an unbroken alignment between the corresponding sequences in X and Y axes. a) Dot plot between ISO1 to ISO1; b) dot plot between ISO1 sequence to its corresponding region in A1 (A1.3L:7601446-7636670); c) dot plot between ISO1 sequence and its corresponding sequence in A7 (A7.3L:7735063-7774259). The mis-annotations designate mutations in A7 and A1 as tandem array size increase and non-TE insertions . As evidenced here, both A7 and A1 possess more sequence compared to their counterpart in ISO1. Thus mis-annotations identify the mutations correctly, but the inferred insertion coordinates are off by few hundred bases.

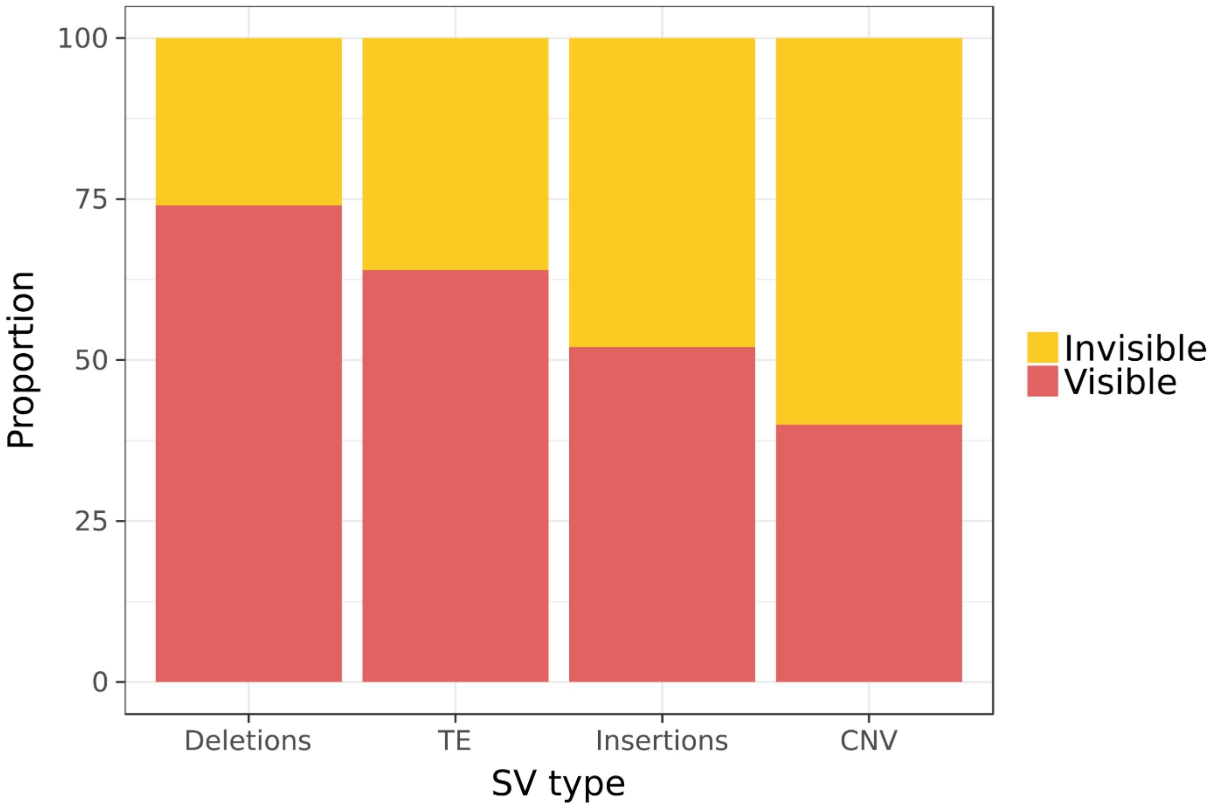

Supplementary Figure 6: Proportion of various large (>100bp) structural variants that are hidden from the methods relying on paired end short reads. Deletions are non-TE sequence deletions, insertions are non-TE sequence insertions, TEs are insertions of transposable element sequences, CNVs represent the increase in sequence copy number.

a)

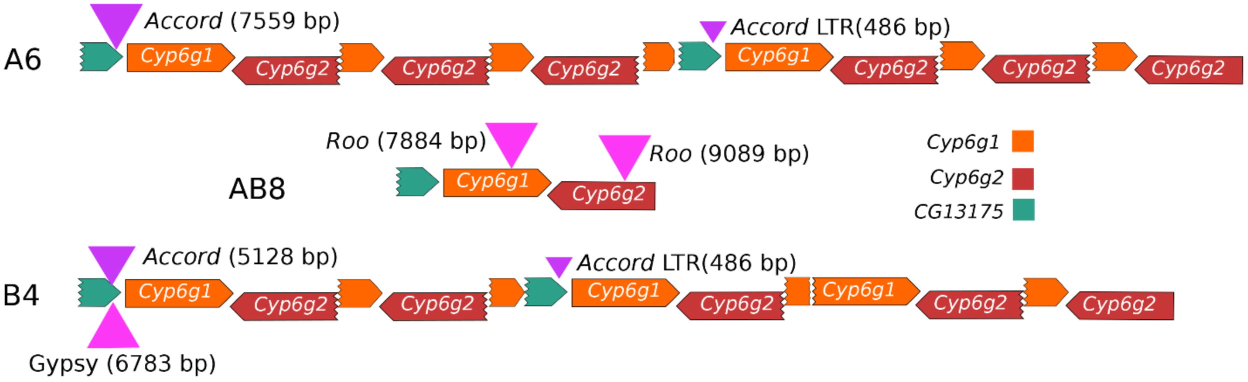

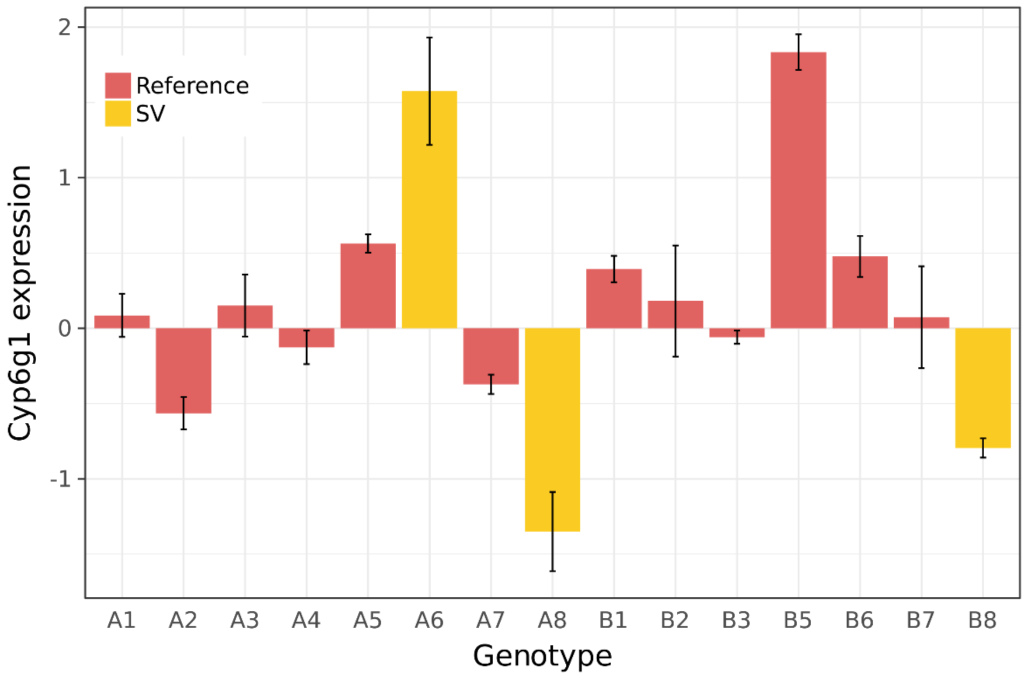

b)

Supplementary Figure 7. a) New *Cyp6g1* alleles uncovered by the assembly of the A6, AB8, and B4 genomes. A6 possesses a full length Accord insertion upstream of *Cyp6g1*. An Accord LTR fragment[3,4] is also inserted upstream of the *Cyp6g1* copy. The AB8 allele has *Roo* insertions in the last exons of the single copy *Cyp6g1* and *Cyp6g2*, presumably disrupting these two genes. B4 contains a 5Kb *Accord* and a 6.7 Kb *Gypsy* insertion in the same position where A6 has the full length Accord insertion. B4 also possess an *Accord* LTR in the same position as A6. b) *Cyp6g1* expression level in female heads for A6 and AB8 (A8 and B8) genotypes are among the highest and lowest *Cyp6g1* expression levels in the RILs. This is consistent with their SV genotypes.

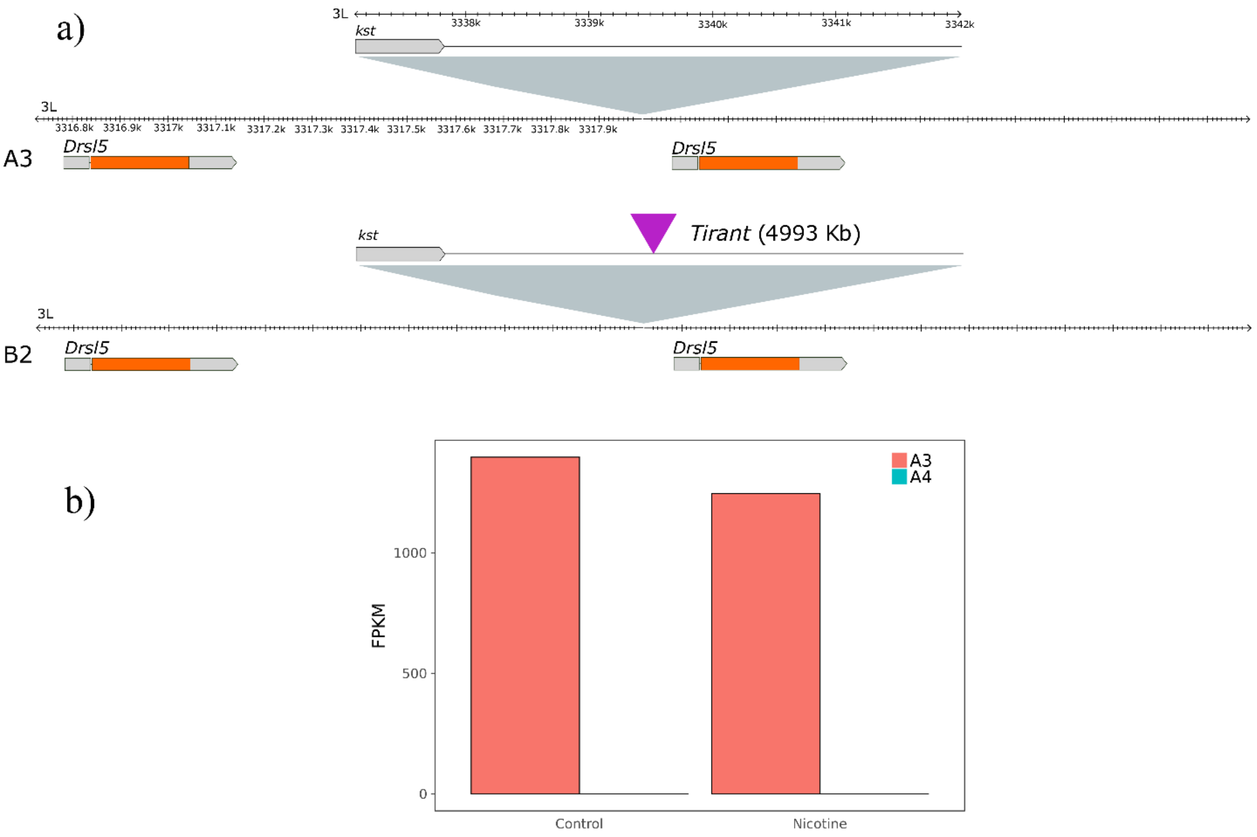

Supplementary Figure 8. a) Duplication alleles of *Drsl5* in A3 and B2. The spacer sequence is derived from the first exon and intron of the gene *Kst* and likely harbors enhancer sequences[5,6]*.* The spacer sequence in B2 also contains a 5 Kb Tirant LTR retrotransposon. B) *Drsl5* expression in A3 is very high but nearly absent in A4 which possesses only one *Drsl5* copy.

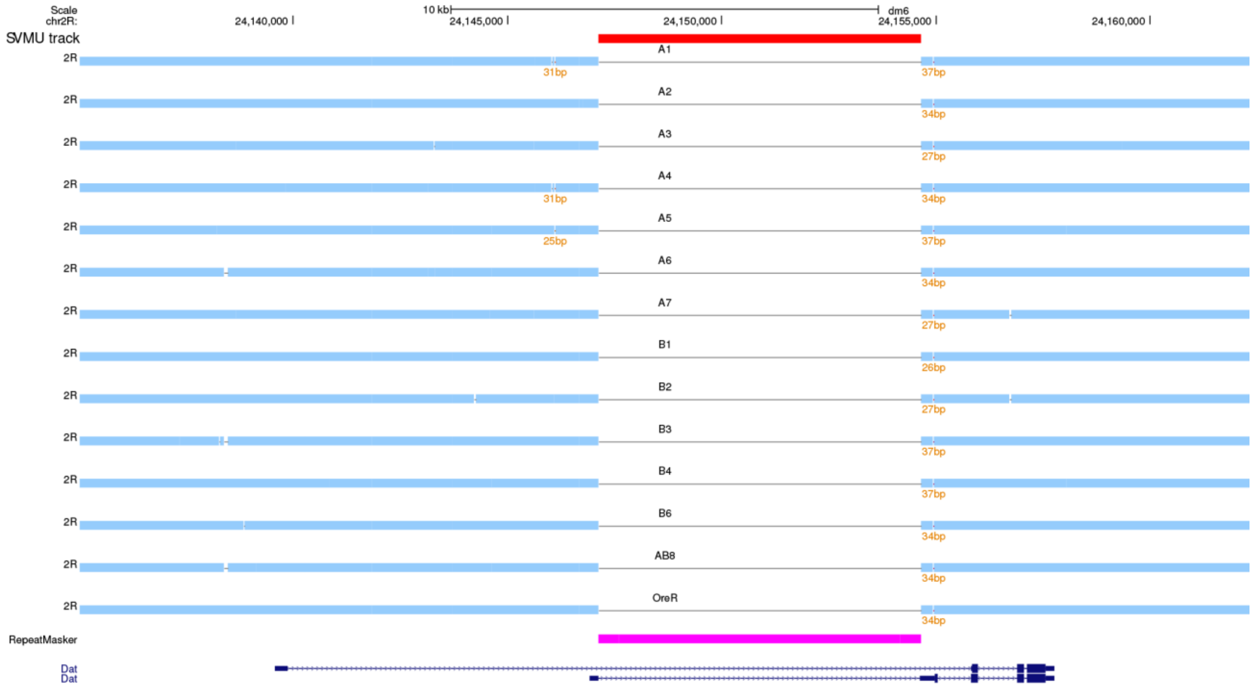

Supplementary Figure 9. *A*ssembly alignment of DSPR founders to the reference strain ISO1 showing insertion of a retrotransposon *412* in the gene *Dat* that causes the visible mutation speck (sp^1) in ISO1. The *de novo* assemblies described here enable discovery of such mutations simply either by looking at the UCSC browser representation of the multiple genome alignment as displayed here[7] (gap corresponding to the pink bar), or by searching through the VCF file (displayed as red bar in the SVMU track).

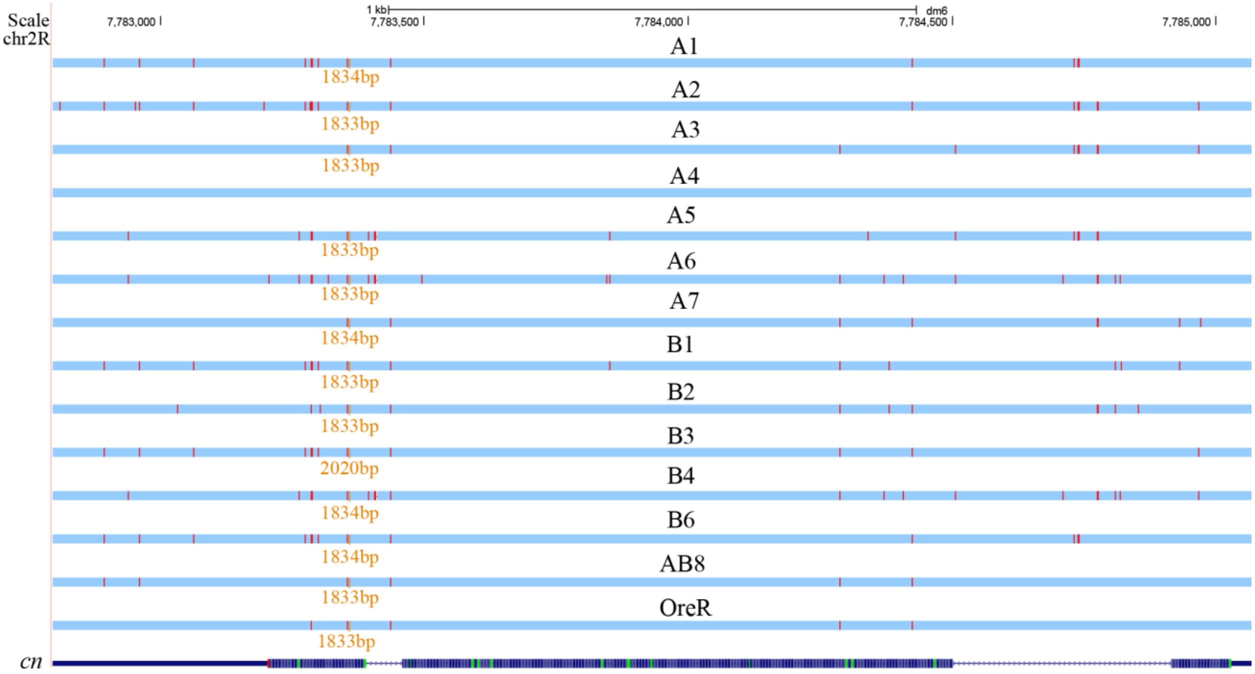

Supplementary Figure 10. Multiple genome alignment of the *de novo* assemblies viewed through UCSC genome browser reveals deletion of a 1.8Kb segment of the 3rd exon of the *Cinnabar* gene in the reference strain ISO1 and A4. This deletion underlies the classical mutation cn_1^8 and causes bright red eye color. The assemblies provide convenient means of identifying the molecular nature of the A4 eye color mutation.

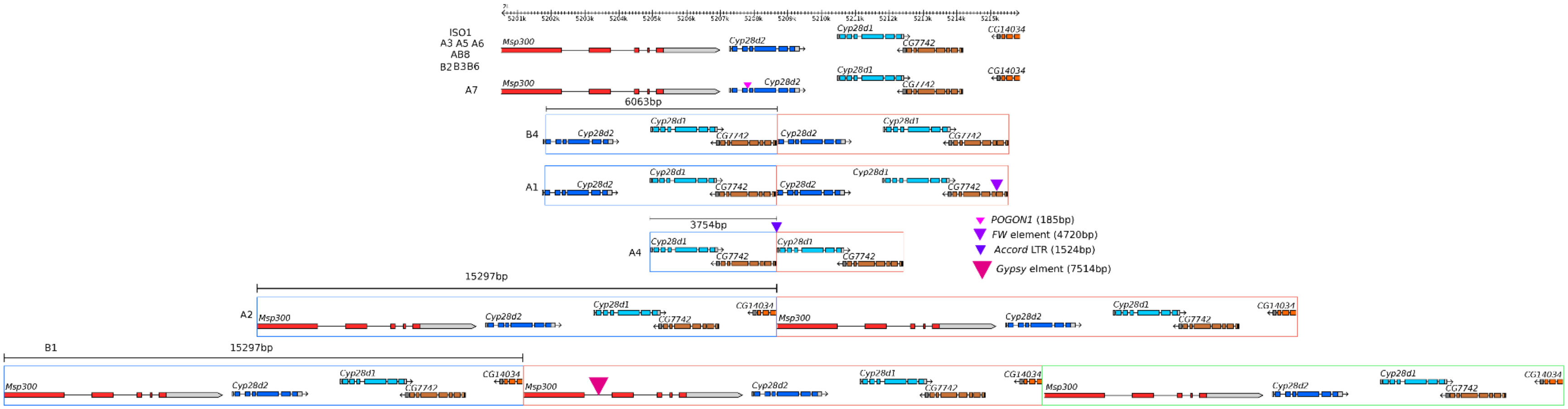

Supplementary Figure 11. Different SV alleles at Cyp28d genes. The POGON1 element in A7 is inserted within an intron of *Cyp28d2*, whereas the FW element in A1 is inserted within an exon of the second *Cyp28d1* copy. The second sequence copy of A1 and B4 is missing part of the first exon of *Cyp28d2*. As evidenced here, B1 consists of three copies of the same 15kb segment that is duplicated in A2, along with a 7.5kb Gypsy insertion in the second copy. Although the nicotine resistance data for RILs carrying the B1 allele are not available[9], the similarity of the genomic region copied in B1 and A2 suggest that RILs with B1 genotype at this locus could be as resistant to nicotine as the A2 genotype RILs.

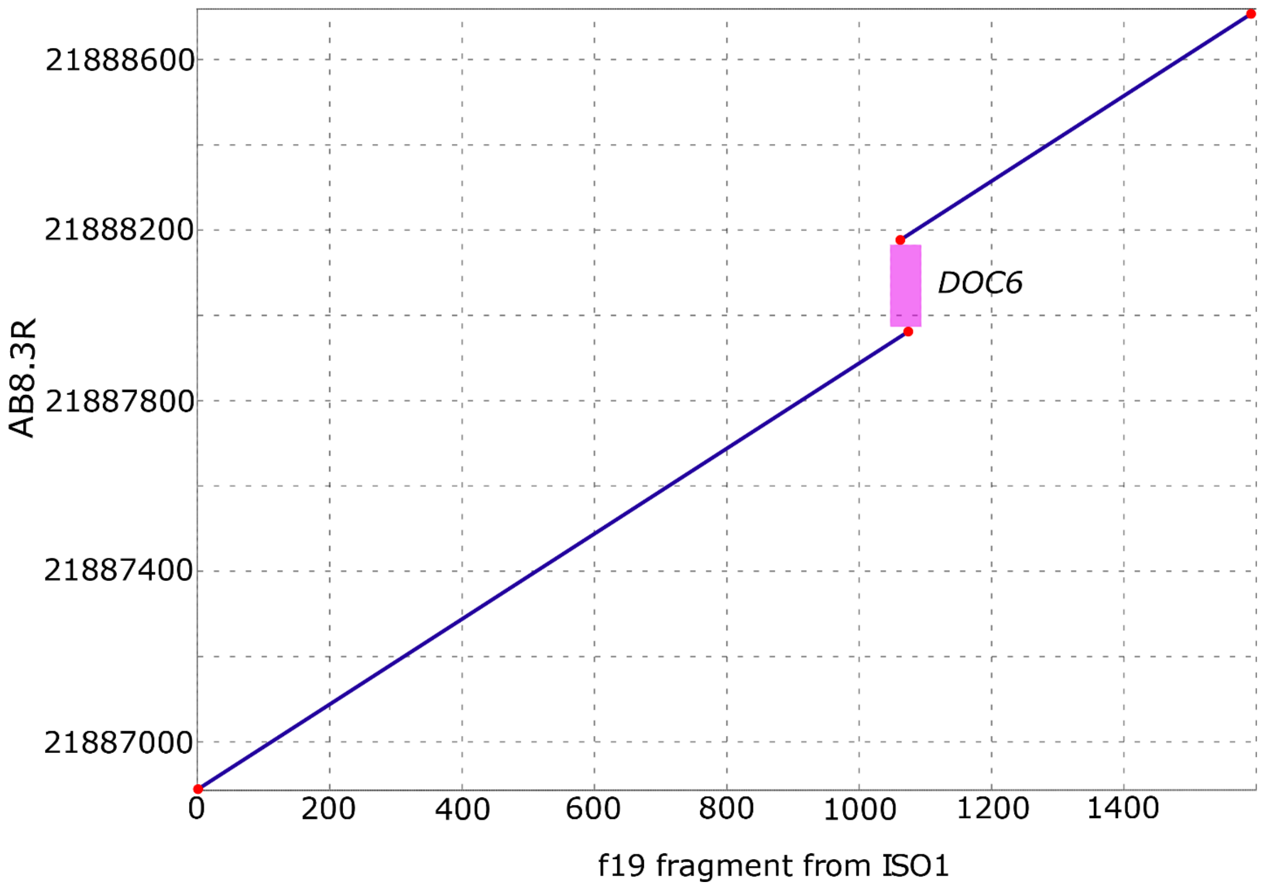

Supplementary Figure 12. Alignment dot plot showing *InR* intronic enhancer fragment named f19 (see [10]) which is disrupted in AB8 by a *DOC6* fragment insertion.

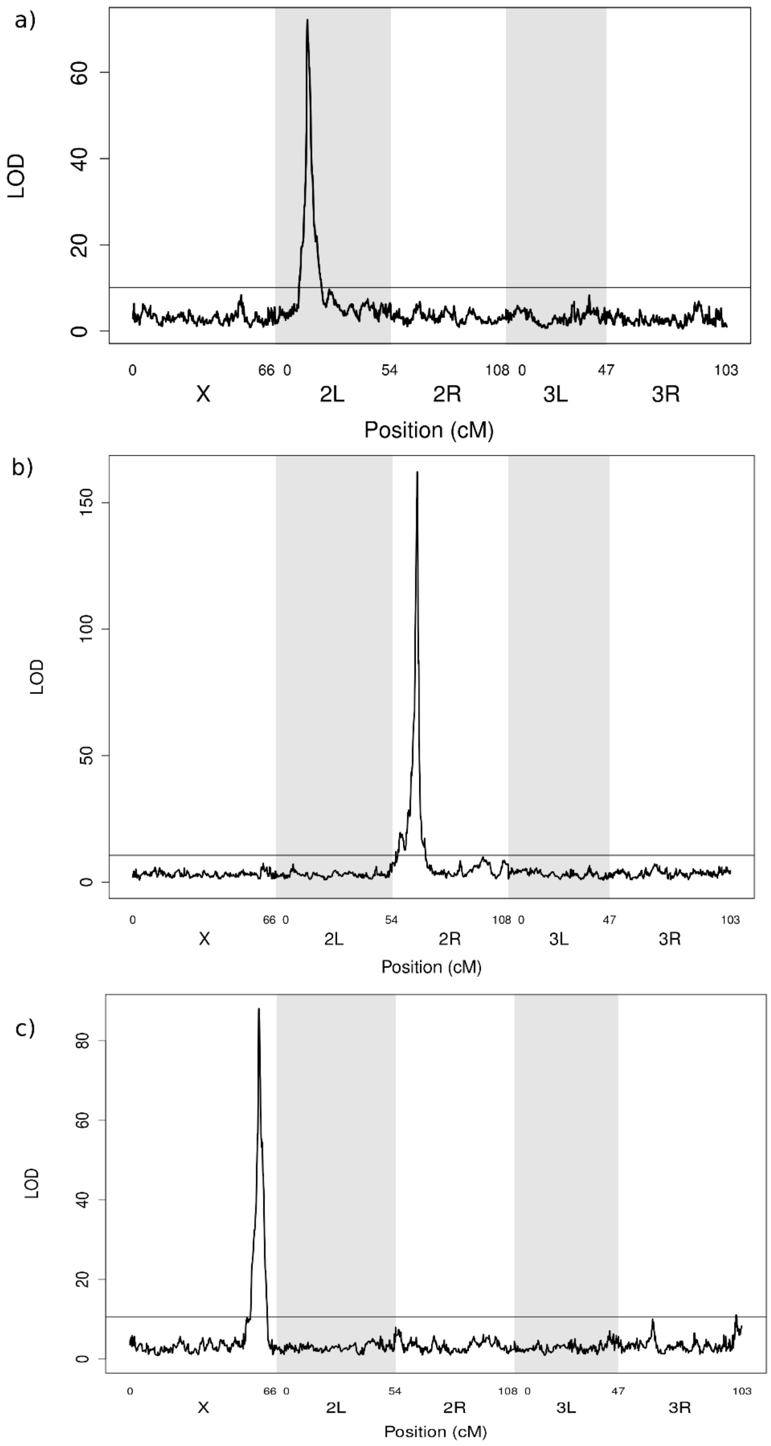

Supplementary Figure 13. Cis-eQTL for a) *Cyp28d1*, b) *Cyp6g1*, and c) *Gss1* transcript level in the females of F1 hybrids between panel A and panel B RILs from DSPR[11,12]. For all of these genes, gene duplications are correlated with high expression in female heads. Similar phenomenon is also observed for other detoxification genes in *Drosophila*[13].

	Arthropoda (Total BUSCO = 1066)				Diptera (Total BUSCO = 2799)			
	Single (%)	Dup (%)	Frag(%)	Miss (%)	Single (%)	Dup (%)	Frag(%)	Miss (%)
**ISO1**	99.0	0.8	0.0	0.2	98.1	0.5	0.8	0.6
**A1**	98.7	1.0	0.1	0.2	98	0.7	0.8	0.5
**A2**	97.3	2.3	0.1	0.3	95.9	2.1	1.2	0.8
**A3**	98.9	0.8	0.0	0.3	98.1	0.6	0.6	0.7
**A4**	98.4	1.4	0.0	0.2	97.7	0.9	0.7	0.7
**A5**	98.9	0.8	0.0	0.3	97.9	0.5	0.9	0.7
**A6**	98.4	1.3	0.0	0.3	98.0	0.6	0.8	0.6
**A7**	95.3	4.4	0.0	0.3	94.9	4.0	0.5	0.6
**AB8**	98.7	1.0	0.0	0.3	98.0	0.6	0.7	0.7
**B1**	98.4	1.4	0.0	0.2	97.8	0.9	0.6	0.7
**B2**	98.8	0.9	0.0	0.3	98.0	0.7	0.6	0.7
**B3**	99.0	0.8	0.0	0.2	98.2	0.4	0.7	0.7
**B4**	98.7	1.0	0.0	0.3	98.3	0.5	0.7	0.5
**B6**	98.9	0.8	0.1	0.2	97.8	0.7	0.8	0.7
**Oregon-R**	98.7	1.1	0.0	0.2	97.6	1.0	0.8	0.6

Supplementary Table 1. Arthropoda and Diptera BUSCO statistics for the sequenced and reference genomes[14,15]. (Dup = duplicated BUSCO, Frag = fragmented BUSCO, Miss = Missing BUSCO).

Chromosome arm	Start	End
X	277911	18930000
2L	82455	19570000
2R	8860000	24684540
3L	158639	18438500
3R	9497000	31845060

Supplementary Table 2. Boundary of euchromatic genomic regions examined in this study(release 6 coordinates[16]).

Supplementary References

1 Chang, C.-H. & Larracuente, A. M. Heterochromatin-enriched assemblies reveal the sequence and organization of the Drosophila melanogaster Y chromosome. *bioRxiv* (2018).

2 dos Santos, G. *et al.* FlyBase: introduction of the Drosophila melanogaster Release 6 reference genome assembly and large-scale migration of genome annotations. *Nucleic Acids Res* **43**, D690-697, doi:10.1093/nar/gku1099 (2015).

3 Schmidt, J. M. *et al.* Copy number variation and transposable elements feature in recent, ongoing adaptation at the Cyp6g1 locus. *PLoS Genet* **6**, e1000998, doi:10.1371/journal.pgen.1000998 (2010).

4 Chung, H. *et al.* Cis-regulatory elements in the Accord retrotransposon result in tissue-specific expression of the Drosophila melanogaster insecticide resistance gene Cyp6g1. *Genetics* **175**, 1071-1077, doi:10.1534/genetics.106.066597 (2007).

5 mod, E. C. *et al.* Identification of functional elements and regulatory circuits by Drosophila modENCODE. *Science* **330**, 1787-1797, doi:10.1126/science.1198374 (2010).

6 Negre, N. *et al.* A cis-regulatory map of the Drosophila genome. *Nature* **471**, 527-531, doi:10.1038/nature09990 (2011).

7 Kent, W. J. *et al.* The human genome browser at UCSC. *Genome Res* **12**, 996-1006, doi:10.1101/gr.229102 (2002).

8 Warren, W. D., Palmer, S. & Howells, A. J. Molecular characterization of the cinnabar region of Drosophila melanogaster: identification of the cinnabar transcription unit. *Genetica* **98**, 249-262 (1996).

9 Marriage, T. N., King, E. G., Long, A. D. & Macdonald, S. J. Fine-mapping nicotine resistance loci in Drosophila using a multiparent advanced generation inter-cross population. *Genetics* **198**, 45-57 (2014).

10 Wei, Y. *et al.* Complex cis-regulatory landscape of the insulin receptor gene underlies the broad expression of a central signaling regulator. *Development* **143**, 3591-3603, doi:10.1242/dev.138073 (2016).

11 King, E. G. *et al.* Genetic dissection of a model complex trait using the Drosophila Synthetic Population Resource. *Genome research* **22**, 1558-1566 (2012).

12 King, E. G., Sanderson, B. J., McNeil, C. L., Long, A. D. & Macdonald, S. J. Genetic dissection of the Drosophila melanogaster female head transcriptome reveals widespread allelic heterogeneity. *PLoS Genet* **10**, e1004322, doi:10.1371/journal.pgen.1004322 (2014).

13 Chakraborty, M. & Fry, J. D. Parallel Functional Changes in Independent Testis-Specific Duplicates of Aldehyde dehydrogenase in Drosophila. *Mol Biol Evol* **32**, 1029-1038, doi:10.1093/molbev/msu407 (2015).

14 Simao, F. A., Waterhouse, R. M., Ioannidis, P., Kriventseva, E. V. & Zdobnov, E. M. BUSCO: assessing genome assembly and annotation completeness with single-copy orthologs. *Bioinformatics* **31**, 3210-3212, doi:10.1093/bioinformatics/btv351 (2015).

15 Waterhouse, R. M. *et al.* BUSCO applications from quality assessments to gene prediction and phylogenomics. *Mol Biol Evol*, doi:10.1093/molbev/msx319 (2017).

16 Hoskins, R. A. *et al.* The Release 6 reference sequence of the Drosophila melanogaster genome. *Genome research* **25**, 445-458 (2015).
