# Supplementary material for: Structural variants exhibit widespread allelic heterogeneity and shape variation in complex traits
Source: Nat Commun. 2019 Oct 25;10:4872. doi: 10.1038/s41467-019-12884-1 (PMC6814777; doi:10.1038/s41467-019-12884-1)
Supplement: Supplementary file 3 — Description of Additional Supplementary Files [file 41467_2019_12884_MOESM3_ESM.pdf]

Description of Additional Supplementary Files

File Name: Supplementary Data 1
Description: List of QTL candidate genes used in the enrichment analysis.

File Name: Supplementary Data 2
Description: *D. melanogaster* gene length summary, SV enrichment, and SV density.

File Name: Supplementary Data 3
Description: Summary of the Pacific Biosciences reads and assembly metrics.

File Name: Supplementary Data 4
Description: Gene length bins used in Figure 2a.
