# Supplementary material for: Structural variants exhibit widespread allelic heterogeneity and shape variation in complex traits
Source: Nat Commun. 2019 Oct 25;10:4872. doi: 10.1038/s41467-019-12884-1 (PMC6814777; doi:10.1038/s41467-019-12884-1)
Supplement: Supplementary file 8 — Reporting Summary [file 41467_2019_12884_MOESM8_ESM.pdf]

nature research

Corresponding author(s): Mahul Chakraborty, Anthony D. Long

Last updated by author(s): Aug 21, 2019

Reporting Summary

Nature Research wishes to improve the reproducibility of the work that we publish. This form provides structure for consistency and transparency in reporting. For further information on Nature Research policies, see Authors & Referees and the Editorial Policy Checklist.

Statistics

For all statistical analyses, confirm that the following items are present in the figure legend, table legend, main text, or Methods section.

n/a	Confirmed	
☐	☒	The exact sample size (*n*) for each experimental group/condition, given as a discrete number and unit of measurement
☐	☒	A statement on whether measurements were taken from distinct samples or whether the same sample was measured repeatedly
☐	☒	The statistical test(s) used AND whether they are one- or two-sided<br>*Only common tests should be described solely by name; describe more complex techniques in the Methods section.*
☐	☒	A description of all covariates tested
☐	☒	A description of any assumptions or corrections, such as tests of normality and adjustment for multiple comparisons
☐	☒	A full description of the statistical parameters including central tendency (e.g. means) or other basic estimates (e.g. regression coefficient) AND variation (e.g. standard deviation) or associated estimates of uncertainty (e.g. confidence intervals)
☐	☒	For null hypothesis testing, the test statistic (e.g. *F*, *t*, *r*) with confidence intervals, effect sizes, degrees of freedom and *P* value noted<br>*Give P values as exact values whenever suitable.*
☐	☒	For Bayesian analysis, information on the choice of priors and Markov chain Monte Carlo settings
☐	☒	For hierarchical and complex designs, identification of the appropriate level for tests and full reporting of outcomes
☐	☒	Estimates of effect sizes (e.g. Cohen's *d*, Pearson's *r*), indicating how they were calculated

Our web collection on statistics for biologists contains articles on many of the points above.

Software and code

Policy information about availability of computer code

Data collection	SMRTanalysis v2.3
Data analysis	Canu v1.3, DBG2OLC v1.0,quickmerge v0.2, DSPRqtl,SVMU v0.2,mscaffolder,Pindel,Pecnv

For manuscripts utilizing custom algorithms or software that are central to the research but not yet described in published literature, software must be made available to editors/reviewers. We strongly encourage code deposition in a community repository (e.g. GitHub). See the Nature Research guidelines for submitting code & software for further information.

Data

Policy information about availability of data

All manuscripts must include a data availability statement. This statement should provide the following information, where applicable:

- Accession codes, unique identifiers, or web links for publicly available datasets
- A list of figures that have associated raw data
- A description of any restrictions on data availability

All scaffolded assemblies and the raw data (HDF5 files and their respective metadata) have been deposited in NCBI under the Bioproject accession PRJNA418342 [https://www.ncbi.nlm.nih.gov/bioproject/?term=PRJNA418342]. All raw SV outputs, and processed data are available at https://github.com/mahulchak/dspr-asm

Field-specific reporting

Please select the one below that is the best fit for your research. If you are not sure, read the appropriate sections before making your selection.

☒ Life sciences ☐ Behavioural & social sciences ☐ Ecological, evolutionary & environmental sciences

For a reference copy of the document with all sections, see nature.com/documents/nr-reporting-summary-flat.pdf

Life sciences study design

All studies must disclose on these points even when the disclosure is negative.

Sample size	Sample size for QTL mapping experiments were determined based on the available phenotype data for the recombinant inbred lines. For the enrichment analysis, the sample size was based on the available QTL mapping experiments conducted with DSPR.
Data exclusions	The Gst gene array was excluded from the enrichment analysis because it presented a complex, irresolvable situation. However, inclusion or exclusion of the gene array led to the same conclusion.
Replication	We performed the candidate gene enrichment Monte Carlo sampling 100000 times to estimate the SV enrichment in the QTL candidate genes.
Randomization	Our study did not involve any experiment that needed randomized allocation of samples to groups.
Blinding	To avoid bias in selecting the candidate genes in our enrichment analysis, we used criteria used by the authors of the original QTL studies to designate the QTL candidate genes.

Reporting for specific materials, systems and methods

We require information from authors about some types of materials, experimental systems and methods used in many studies. Here, indicate whether each material, system or method listed is relevant to your study. If you are not sure if a list item applies to your research, read the appropriate section before selecting a response.

Materials & experimental systems

n/a	Involved in the study	
☒	☐	Antibodies
☒	☐	Eukaryotic cell lines
☒	☐	Palaeontology
☐	☐	Animals and other organisms
☒	☐	Human research participants
☒	☐	Clinical data

Methods

n/a	Involved in the study	
☒	☐	ChIP-seq
☒	☐	Flow cytometry
☒	☐	MRI-based neuroimaging

Animals and other organisms

Policy information about studies involving animals; ARRIVE guidelines recommended for reporting animal research

Laboratory animals	Females of Drosophila melanogaster strains from Drosophila Synthetic Population Resource and Oregon-R
Wild animals	None
Field-collected samples	No field-collected sample was used.
Ethics oversight	Flies were treated following the guidelines set by University of California Irvine.

Note that full information on the approval of the study protocol must also be provided in the manuscript.
